# Histogram analysis based on unenhanced CT for identifying thymoma and lymphoma among prevascular mediastinal incidentalomas

**DOI:** 10.1186/s40644-023-00617-z

**Published:** 2024-01-04

**Authors:** Ming Liu, Yang Zhang, Li-Heng Liu

**Affiliations:** 1 Department of Radiation Oncology, Shanghai Pulmonary Hospital, Tongji University School of Medicine, Shanghai, China; 2 Department of Radiology, Dongying People’s Hospital, Shandong, China; 3 Department of Radiology, Zhongshan Hospital, Fudan University, No. 180 Fenglin Road, Shanghai, China; 4 Shanghai Institute of Medical Imaging, Shanghai, China

**Keywords:** Thymic epithelial tumors, Histogram analysis, Tomography, X-ray computed, Differential diagnosis

## Abstract

**Objective:**

To determine whether histogram analysis based on unenhanced CT can play a role in the differential diagnosis of thymoma and lymphoma from thymic hyperplasia and cyst (mean CT attenuation > 10 HU).

**Materials and methods:**

This retrospective study included consecutive asymptomatic participants who have prevascular mediastinal lesions incidentally detected by unenhanced CT between December 2013 and August 2020, and with definitive diagnosis by pathology or additional radiologic work-ups. A total of thirteen histogram parameters on enhanced CT were calculated for each lesion, then were compared between tumor (thymoma + lymphoma) and non-tumor (hyperplasia + cyst). Receiver operating characteristic analysis was conducted to investigate the performance of histogram parameter for identifying tumor.

**Results:**

The study population included 192 patients (106 men and 86 women) with a mean age of 50.5 years at the time of CT examination. Of them, 94 patients have tumor (87 thymomas and 7 lymphoma) and 98 have non-tumor (48 thymic hyperplasia and 50 cysts). Nine of the thirteen histogram parameters revealed significant difference between the two groups, including median, minimum, range, 10th percentile, 90th percentile, kurtosis, skewness, uniformity and entropy. No significant difference was observed in the mean CT attenuation between groups. Higher median was found to be independent predictors for distinguishing tumor from non-tumor, and can achieve an area under the curve (AUC) of 0.785 (95% confidence interval [95% IC], 0.720–0.841).

**Conclusions:**

Histogram analysis based on unenhanced CT may be able to provide some help in the differential diagnosis of incidental lesions in prevascular mediastinal.

**Grand support:**

This study was sponsored by Natural Science Foundation of Shanghai (No. 21ZR1459700).

## Introduction

The widespread application of chest computed tomography (CT) has led to the increased detection of incidental prevascular mediastinal lesions. In people without known malignancy, the most likely pathologic conditions of these incidental lesions include thymic hyperplasia, thymic cyst, thymoma and lymphoma [1–4]. For these incidentaloma patients, the radiologic work-up should aim at establishing whether the lesion is tumor or not. Because further diagnosis and treatment strategies including complete surgical resection are recommended for tumor, while thymic hyperplasia and cyst do not require follow-up [3, 5].

However, most lesions are found on unenhanced CT, which was performed for reasons such as pulmonary nodules screening, rather than suspicion of thymic disease[1; 6]. Depend on unenhanced CT, it is difficult to distinguish thymoma and lymphoma from thymic hyperplasia with atypical shape, or from cyst with nonwatery attenuation. Previous studies have reported an unnecessary thymectomy rate of 26% which was considered mainly attributed to the inaccurate radiologic diagnosis[7, 8]. For the indeterminate lesions, further radiologic work-up including enhanced CT, chemical shift and enhanced MRI and even PET are required[9, 10]; this inevitably leads to additional medical costs and radiation exposure. Thus, accurate and practical approach based on initial unenhanced CT to distinguish tumor from non-tumor in incidental thymic lesions remains an unmet clinical requirement.

Although there is significant overlap in mean attenuation, the grayscale distribution and heterogeneity within the lesion may varies across the aforementioned disease entities, as they have different internal components. Histogram analysis of medical imaging has been confirmed to allow sensitive and accurate detection of grayscale distribution between different disease entities [11]. Therefore, we hypothesized that histogram analysis based on unenhanced CT could play a role in the differential diagnosis of thymoma and lymphoma from thymic hyperplasia and cyst. In addition, histogram analysis is easy to obtain in clinical practice, and can be conducted in the embedded post-processing module of the picture archiving and communication system (PACS) in many hospitals. It’s another reason we preferred to apply histogram analysis instead of other more complicated artificial intelligence methods in this study.

The purpose of this study was to determine whether histogram analysis based on unenhanced CT can play a role in the differential diagnosis of tumor (thymoma + lymphoma) from non-tumor (hyperplasia + cyst) in prevascular mediastinal incidentalomas.

## Materials and methods

This study was approved by local institutional review board and informed consent was waived according to the retrospective design.

### Study sample

The medical database was queried for all consecutive asymptomatic participants (age > 20 years) who was found with a solitary prevascular mediastinal lesion by unenhanced chest CT as part of their health examination from December 2013 to August 2020. Inclusion criteria were as follows: (1) largest diameter of the lesion ≤ 40 mm; (2) have a definitive pathologic diagnosis of thymic hyperplasia, cyst or tumor though thymectomy or needle biopsy, or have a definitive clinical diagnosis of thymic hyperplasia or cyst by further MRI examination and follow-up. Exclusion criteria were as follows: (1) with malignancy history before CT examination; (2) the lesion with a typical shape of thymus, such as butterfly wing or triangle; (3) with mean CT attenuation ≤ 10 Hounsfield Units (HU); (4) with severe imaging artifact. Finally, we enrolled 192 patients in this study.

### CT Image Acquisition

All unenhanced chest CT images was acquired using 3 different CT scanners with ≥ 128 detector rows (Somatom Definition AS + 128, Siemens, Germany; or Erlangen Brilliance 128, Philips, Best, The Netherlands; or uCT760, United Imaging, Shanghai, China). All unenhanced scans were performed in the supine position with 120 kVp, 80–200 mAs. The mediastinal-window (level of 40 HU and width of 400 HU) images were reconstructed with a thickness of 5 mm.

### Imaging analysis

**Fig. 1:**
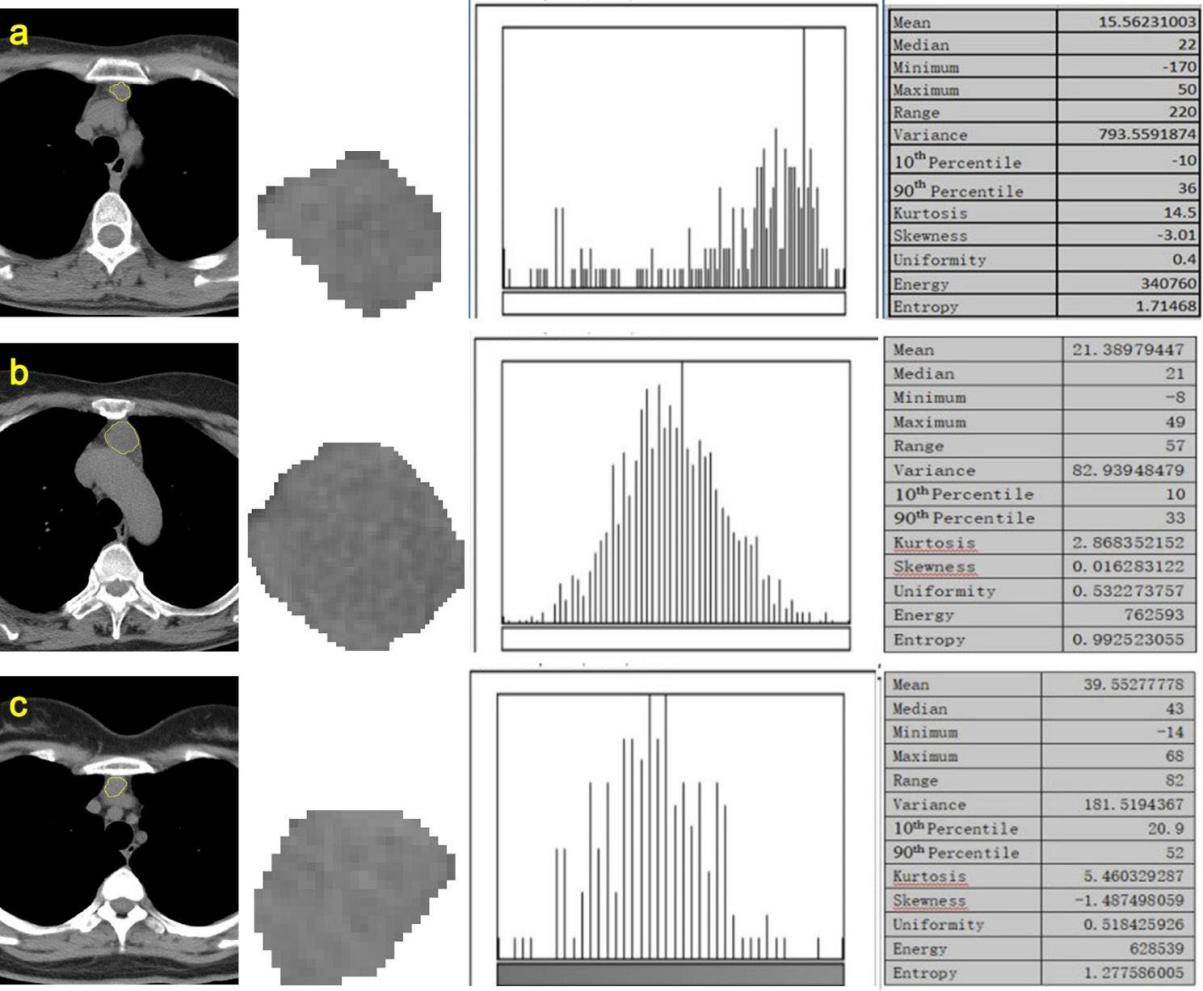
Axial unenhanced CT images, ROI setting, pixel intensity distribution histogram and histogram parameters of representative cases (**a**, thymic hyperplasia; **b**, thymic cyst; **c**, thymoma)

Region of interest (ROI) positioning was performed by a board-certified radiologist with 5 years of experience in chest imaging using an imaging processing software (ImageJ, National Institutes of Health, MD). To test the effect of inter-observer variability in ROI delineation which may affect parameters measurement, another radiologist with 7 years of experience also independently conducted ROI positioning. Both radiologists were blinded to all clinical data and final diagnosis at the time of analysis. The ROI was drawn within the lesion and along the outline, with a freehand shape, at the single slice mediastinal image that showing the largest lesion-diameter (Fig. 1). The determined ROIs were transferred into the preexisting Python Pyradiomics Library (version 2.0.1; available at https://github.com/Radiomics/pyradiomics) for histogram analysis. The following thirteen parameters were extracted from each ROI: mean, median, minimum, maximum, range, variance, 10th and 90th percentile, kurtosis, skewness, uniformity, energy and entropy.

### Reference standard

The diagnosis of thymoma and lymphoma was established by histopathologic examination after thymectomy or needle biopsy, while thymic hyperplasia and cyst established by histopathologic examination or definitive clinical diagnosis. The clinical diagnosis of thymic hyperplasia and cyst was determined based on further radiologic work-up including chemical shift MRI, contrast enhanced MRI and/or ^18^ F fluorodeoxyglucose PET.

### Statistical analysis

Statistical analyses were performed using SPSS 22.0 (IBM Corp, NY). Continuous variables are expressed as means ± standard deviations. Histogram parameters were compared between disease entities using the student t-test or the Mann-Whitney test. The potential of histogram parameters for distinguishing thymoma and lymphnoma from thymic hyperplasia and cyst was evaluated by area under the receiver operating characteristic (ROC) curve (AUC). Interobserver variability of texture features extraction was evaluated by using intraclass correlation coefficients (ICC < 0.40, poor agreement; 0.40 ≤ ICC < 0.60, moderate agreement; 0.60 ≤ ICC < 0.80, good agreement; ICC > 0.80, excellent agreement). A P value of < 0.05 was considered indicative of a statistically significant difference.

## Results

### Patient demographics

**Fig. 2:**
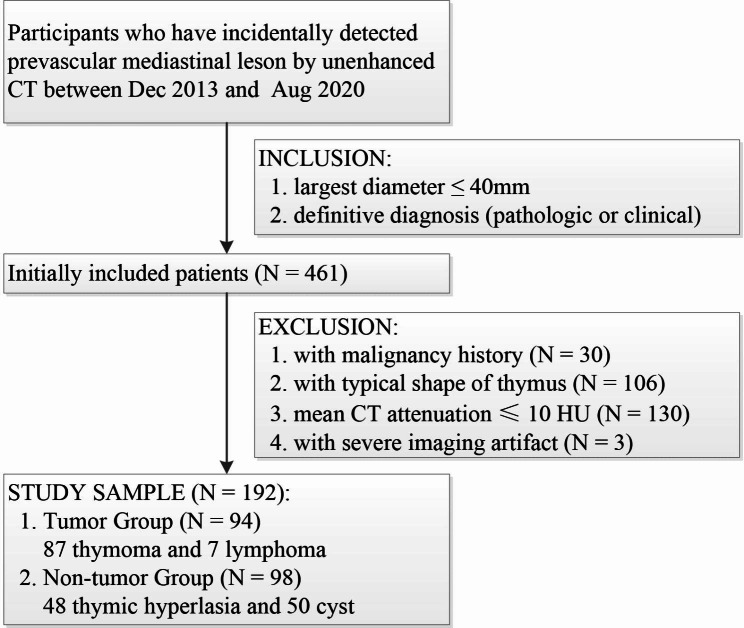
Study flowchart show patients inclusion and exclusion criteria

We initially identified 461 patients who were incidentally found have prevascular mediastinal lesions of definitive diagnosis, and then excluded 269 patients because of the presence of malignancy history (N = 30), lesions’ typical shape (N = 106), with mean CT attenuation ≤ 10 HU (N = 130) or severe imaging artifact (N = 3) (Fig. 2). Thus, the study population included 192 patients (106 men and 86 women) with a mean age of 50.5 years at the time of CT examination. Of the included 192 patients, 87 with thymomas (all confirmed by histology), 7 with lymphoma (all confirmed by histology), 48 with thymic hyperplasia (6 by histology, 42 by clinical diagnosis), and 50 with cysts (11 by histology, 39 by clinical diagnosis). The distribution of basic data is shown in Table 1.

**Table 1:** Patients basic characteristics between groups

Parameter	Thymoma (N = 87)	Lymphoma (N = 7)	Hyperplasia (N = 48)	Cyst (N = 50)	P-vlaue (Thymoma + Lymphoma vs. Hyperplasia + Cyst)
**Age (year)**	54.6 ± 13.1	57.4 ± 14.1	40 ± 11	52.6 ± 12.8	0.236
**Gender**					0.470
**Male**	45	5	25	31	
**Female**	42	2	23	19	
**Max-diameter (mm)**	26.5 ± 12.0	27 ± 8.2	32.3 ± 8.3	20.2 ± 8.4	0.149

### Histogram parameters between thymoma and non-thymoma

**Fig. 3:**
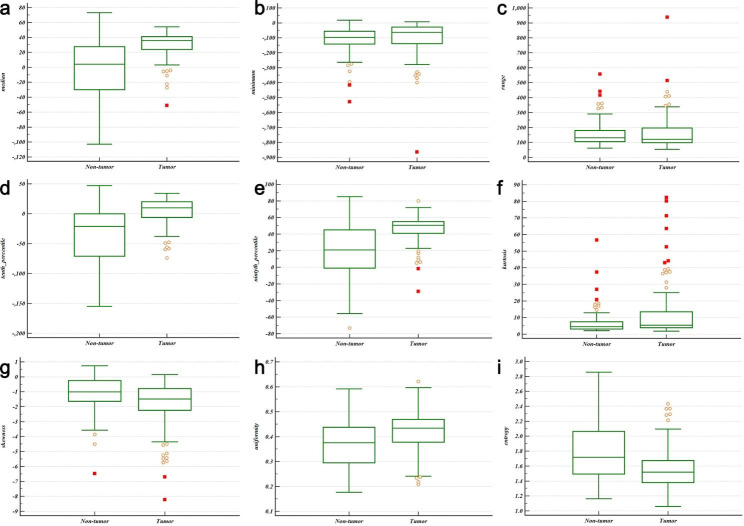
Box-and-whisker plots for distributions of statistically significant parameters between tumor and non-tumor lesions (**a**, median; **b**, minimum; **c**, range; **d**, 10th percentile; **e**, 90th percentile; **f**, kurtosis; **g**, skewness; **h**, uniformity; **i**, entropy)

The histogram parameters between disease entities are summarized in Table 2. No significant difference was observed in age, gender or max-diameter between tumor (N = 94) group and non-tumor (hyperplasia and cyst, N = 98) group (P = 0.236, 0.470 and 0.149, respectively). Of the thirteen histogram parameters, median, minimum, range, 10th percentile, 90th percentile, kurtosis, skewness, uniformity and entropy revealed significant difference between the two groups. Distribution of statistically significant parameters between tumor and non-tumor lesions is shown in Fig. 3.

**Table 2:** Distribution of histogram parameters on unenhanced CT between groups

Parameter	Tumor group (N = 94) (87 thymoma and 7 lymphoma)	Non-tumor group(N = 98) (48 hyperplasia and 50 cyst)	P-vlaue
Mean	14.9 ± 4.3	17.4 ± 7.2	0.061
Median	30.0 ± 18.1	-2.6 ± 37.3	< 0.001
Minimum	-104.2 ± 122.9	-113.9 ± 87.4	0.001
Maximum	64.5 ± 24.1	43.9 ± 32.6	0.075
Range	171.2 ± 125.8	157.9 ± 83.9	0.001
Variance	535.1 ± 704.1	667.6 ± 965.1	0.510
10th Percentile	3.1 ± 23.1	-34.8 ± 43.5	< 0.001
90th Percentile	45.9 ± 16.8	19.1 ± 31.7	< 0.001
Kurtosis	13.2 ± 17.3	7.0 ± 7.6	< 0.001
Skewness	-1.91 ± 1.67	-1.1 ± 1.2	0.001
Uniformity	0.42 ± 0.08	0.37 ± 0.10	0.029
Energy (× 10^6^)	1.5 ± 1.5	1.3 ± 1.8	0.068
Entropy	1.58 ± 0.29	1.8 ± 0.4	0.008

**Fig. 4:**
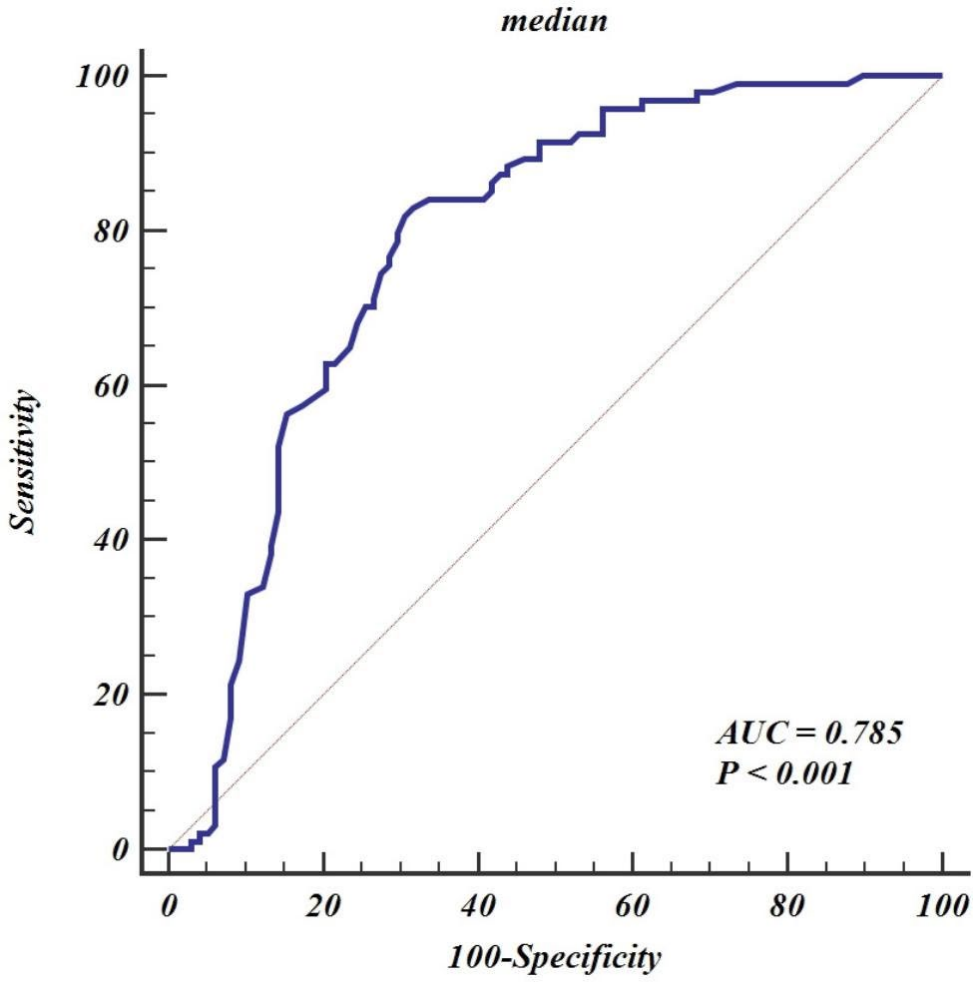
The receiver operating characteristic curve of median. AUC = area under the curve

The parameters with significant difference as well as mean CT attenuation were used as input variables for multiple logistic regression analysis. Higher median was found to be independent predictors for distinguishing tumor from non-tumor and can achieve an AUC of 0.785 (95% confidence interval [95% IC], 0.720–0.841), with sensitivity of 82.9%, specificity of 68.4%, positive predictive value (PPV) of 71.6%, and negative predictive value (NPV) of 80.7% at an optimal cutoff of 17 (Fig. 4). The mean time spent on image-processing is 4.2 ± 2.7 min for one lesion.

### Interobserver variability evaluation

All histogram parameters extracted two sets of ROIs independently determined by two radiologists showed excellent agreement (ICCs range from 0.833 to 0.971).

## Discussion

It is critical but challenging to use unenhanced CT for distinguishing thymoma and lymphoma from thymic hyperplasia with atypical shape, or from cyst with nonwatery attenuation. Our results demonstrate the potential of histogram parameters derived from unenhanced CT for identifying tumor lesions. We found that tumor including thymoma and lymphoma exhibited significantly different histogram parameters, including median, minimum, range, 10th percentile, 90th percentile, kurtosis, skewness uniformity and entropy, than did thymic hyperplasia and cyst. Of these parameters, median has been determined as an independent predictor, and achieved an AUC of 0.785 in diagnosing of tumor.

Our study demonstrates the role of histogram analysis based on unenhanced CT in distinguishing tumor from non-tumor lesions in prevascular mediastinal incidentalomas. The result is in agreement with that of a recent study which used CT histogram and texture to discriminate thymoma and thymic hyperplasia [12]. But the previous study included only thymic hyperplasia as the differential diagnosis from thymoma, and did not include thymic cyst with nonwartery attenuation, which also usually appear similar morphological features with thymoma. In our study, we enrolled a cohort of 192 consecutive patients with incidental prevascular mediastinal lesions that covering all common disease entities (lymphoma, thymic tumors, hyperplasia and cysts). Our research process seems to be more consistent with the real clinical scenarios.

In addition, histogram analysis represents a conventional image post-processing technology, and could be embedded into the PACS for routine clinical application in many institutes. Compared to texture and radiomics analysis, histogram analysis is more accessible in clinical practice, and its parameters have clearer meanings and interpretability. Based on results of many previous studies and its own mathematical model, histogram analysis allows for evaluating the distribution of gray-level of values (CT attenuation) within a given ROI; this can help us to explore the different internal composition between thymoma and non-thymoma lesions, though they share the similar mean CT attenuation on both visual observation and quantitative measurement. In a previous study, Nagayama et al. conducted histogram analysis on CT images in a cohort of 232 patients with indeterminate adrenal lesions, and found histogram parameters could enable differentiation lipid-poor adenomas from non-adenomas [13]. Their findings are in line with ours with respect to the basis of theoretical application of histogram. Both studies demonstrate the feasibility of histogram analysis on enhanced CT in the differentiate diagnosis of thymic or adrenal incidental lesions. The feasibility may be attributed to the common spectrum of disease at the two sites. Of incidentalomas at the two specific sites, differentiate diagnosis and subsequent management largely depend on the composition within the lesion (solid, cysts or fat).

In fact, many incidental lesions in prevascular mediastinal could be safely classified as benign based on their shape and density. About half (236/461) of the lesions in our study were diagnosed as hyperplasia or cyst by unenhanced CT. Histogram analysis could be considered as a further method to distinguish the remaining lesions with atypical shape and nonwartery attenuation.

In the present study, a limitation exists as that histogram analysis was based on a single-slice image at the level of the largest cross section of the tumor, rather than the whole-tumor. However, compared to the whole-volume model analysis, single-slice analysis maybe more convenient, and can be more easily conducted in clinical practice. Second, only seven patients with lymphoma are identified and included in the study population; this does not match the actual incidence of lymphoma, which consist about 25% of anterior mediastinal mass [4]. Several reasons may have contributed to this reality. We conducted this study in the population who undergo lung CT for the purpose of health check-up, and with no known history of malignancy or suspected diseases. In addition, all of the lesions in the present study were incidentally detected and patients did not have any related symptoms. Based on the specially selected study population and enrollment process, the low proportion of lymphoma in the present study may be understandable, even though it does not represent the true incidence. Exactly, certain selection biased exists in the present study, and it definitely has an impact on the generalizability of our findings. Third, the retrospective nature of this study is another major limitation.

## Conclusion

In conclusion, histogram analysis based on unenhanced CT may be able to provide some help in the differential diagnosis of incidental lesions in prevascular mediastinal. Nine parameters including median enabled differentiation of tumor from non-tumor lesions, potentially avoiding the need for additional radiologic work-ups and reducing unnecessary thymectomy in some patients.

## Data Availability

The datasets of this study are available from the corresponding author.

## Abbreviations

CT: Computed tomography
ROI: Region of interest
MRI: Magnetic Resonance Imaging
HU: Hounsfield Units
ROC: The receiver operating characteristic
AUC: Area under the curve
ICC: Intraclass correlation coefficients
PACS: The picture archiving and communication system
